# The Effect of Parental Involvement and Socioeconomic Status on Junior School Students’ Academic Achievement and School Behavior in China

**DOI:** 10.3389/fpsyg.2018.00952

**Published:** 2018-06-19

**Authors:** Wenjie Duan, Yuan Guan, He Bu

**Affiliations:** Wuhan University, Wuhan, China

**Keywords:** parental involvement, home-based involvement, academic socialization, academic achievement, school behavior, socioeconomic status

## Abstract

A survey was conducted on 19,487 Chinese junior school students to elucidate the moderating role of socioeconomic status (SES) in the relationship between parental involvement (i.e., home-based involvement and academic socialization) and junior school students’ performance in school (i.e., academic achievement and school behavior). The data includes 10,042 males and 9,445 females (mean age = 14.52, *SD* = 1.24). It was taken from the 2013–2014 Chinese Educational Panel Survey (CEPS), that was administrated by the National Survey Research Center at Renmin University of China. The results demonstrate that SES negatively moderates both the relationship between academic socialization and academic achievement, and the relationship between home-based involvement and school behavior. Findings imply that parental involvement activities are highly beneficial for junior school students in families with low SES. Academic socialization is generally associated with academic success, whereas home-based involvement closely relates to school behavior. Future home-based interventions can be developed to promote parental involvement activities in low-SES families. The results also showed important implications for the development of family education in China.

## Introduction

Previous studies have shown that parental involvement impacts on the academic achievement and behavior of adolescents (Fan and Chen, 2001; Jeynes, 2016). This brief study aims to promote the development of children and adolescents by examining the relationship between parental involvement, socioeconomic status (SES), and junior school students’ performance (e.g., academic achievement and school behavior). A survey that took a national representative sample for China was considered in this work.

Parental involvement generally includes three aspects: home-based involvement; school-based involvement; and academic socialization (Fan and Chen, 2001; Hill and Chao, 2009; Hill and Tyson, 2009). Home-based involvement entails parents’ involvement activities at home such as supervising homework, checking homework, and talking about school life; school-based involvement includes some activities implemented at school such as communicating with teachers, attending the class meeting, and participating in school activities; academic socialization mainly includes parents’ expectations and faith about their children’s education (Hill and Tyson, 2009; Benner et al., 2016). This framework was usually used in American culture (Wang and Sheikh-Khalil, 2014). For example, using data for 15,240 middle school students in America, Benner et al. (2016) tested the relationship between parental involvement (i.e., home-based involvement, school-based involvement, and academic socialization) and academic achievement. With the American data of Education Longitudinal Study 2002–2013, Day and Dotterer (2018) assessed the connection between parental involvement (i.e., home- and school-based involvement and academic socialization) and academic achievement. However, such framework should be modified in the Chinese context (Lau et al., 2011). By using a data of 310 kindergartens, elementary schools and secondary schools from Home-School Cooperation Committee of the Education Department in Hong Kong, Ng (1999) found that Chinese parents didn’t like to get involved in school, and teachers didn’t like to get parents involved in school either. Using a sample of 431 students in Hong Kong, Lau et al. (2011) demonstrated that when compared with home-based involvement, school-based involvement had less influence on children’s educational performance. In interviews with 30 migrant children (*mean age* = 13) in Zhejiang, China, Fang et al. (2017) found that school-based involvement was less mentioned. In this case, the current studies only focus on home-based involvement and academic socialization in the Chinese context. Previous studies have demonstrated that home-based involvement and academic socialization positively influenced academic achievement and school behavior (Fan and Chen, 2001; Chen and Gregory, 2009; Hill and Tyson, 2009; Benner et al., 2016). For example, Manz et al. (2014) found that a mother’s home-based involvement increased children’s interpersonal skills and decreased the incidence of negative classroom behaviors. Hayes (2012) found that home-based involvement increased adolescents’ academic achievement. Hill and Tyson (2009) further claimed that academic socialization was positively related to academic achievement.

Nevertheless, socioeconomic status significantly affects the relationship between parental involvement and adolescent performance (Stevenson and Baker, 1987; Byun et al., 2012). Parents with low SES typically practice low levels of academic socialization with their children (Carolan and Wasserman, 2015). By contrast, families with high SES usually engage in high-quality activities of home-based involvement (Fantuzzo et al., 2004). For instance, Conger and Donnellan (2007) found that parents with high SES had better communication with their children. In an expansion study on children’s communicative-pragmatic ability, with a sample of 390 Italian-speaking children (Bosco et al., 2013) found that family SES has small yet significant positive effect on children’s pragmatic ability, and the effect was still existed during the middle part of their childhood. In addition, other studies suggested that SES is linked with the academic achievement of adolescents (Hill and Tyson, 2009; Byun et al., 2012). Adolescents from families with high SES tend to display good academic achievement (Sirin, 2005; Reardon, 2011).

According to the theory of cultural reproduction, a high SES family provides more educational resources to their offspring, and promotes adolescents’ educational achievement (Bourdieu, 1973; Bourdieu and Passeron, 1990). Coleman (1988) considered that a family with high SES can provide a better living environment and more educational resources for their child or children. For example, with a longitudinal data of 2744 adolescents, Morris et al. (2018) found that children from low SES families tended to live in low SES neighborhoods, causing a higher tendency for them to take up smoking. With the Independent Freshman Admission administrative data from an elite university (i.e., Peking University in China), Liu et al. (2014) found that adolescents in high SES families had more chances to pass the selection process to enter these universities. In such circumstances, with less social capital, parental involvement is more important for adolescents in low SES families. According to the cultural mobility model, Dimaggio (1982) argued that a low SES environment acted as an incentive for parents to invest in their children to make up for other factors that disadvantaged them. Parental involvement acts as a support mechanism for children, whereas adolescents in high SES families had better living conditions and more educational resources. This meant that the effect of parental involvement was greatly reduced. Thus, parental involvement is more effective for adolescents in low SES families than for adolescents in high SES families.

The preceding literature review implies that the moderating role of SES varies among different aspects of parental involvement (i.e., home-based involvement and academic socialization) and adolescent school performance (i.e., academic achievement and school behavior). For instance, with the data taken from National Education Longitudinal Study 88-94, Kim and Schneider (2005) further claimed that adolescents from low-SES families benefited more from parental involvement in academic activities. With the data from the National Longitudinal Survey of Youth-Children and Young Adults in America, Jaeger (2011) found that socioeconomic status played a moderating role in the relationship between cultural capital (which contained partial content of parental involvement) and adolescent academic achievement. Using data from 10 public high schools in America, Wang and Sheikh-Khalil (2014) found that home-based involvement was more strongly correlated with school academic behavior in low-SES families. However, both Wang and Sheikh-Khalil (2014) and Jaeger (2011) tested the moderation effect of family SES in the American context. Yet few studies have tested the moderating effect of SES between parental involvement and adolescent performance in other specific culture, such as in the Chinese culture.

As a result, we hypothesize that socioeconomic status negatively moderates the relationship between parental involvement and junior school students’ performance in the Chinese context. Data from China Education Panel Study of 2013–2014 would be used to validate this hypothesis. The meaning of the current study may be as follows. First, the current study highlights the importance of parental involvement and may serve to upgrade the importance of family education in China. Second, the current study can be regarded as a suggestion for a family intervention project to focus on promoting parental involvement.

## Materials and Methods

### Participants and Procedures

Data for the present study was adopted from the Chinese Educational Panel Survey 2013–2014 (CEPS), which was conducted by the National Survey Research Center at Ren-min University of China. CEPS 2013-2014 is available openly (CEPS, 2015). CEPS is a nationally representative survey. Data collecting procedures were designed in multi-stage stratified probability proportional sampling (PPS). Four stages were included: 438 classes in 112 schools from 28 country level units were sampled in probability proportionality, and all of the junior school students in the sampled classes were selected (CEPS, 2014). The data comprised 19,487 students (10,042 males and 9,445 females; mean age = 14.52, *SD* = 1.24). According to the implementation report of CEPS, the valid response rate was 100%. A human ethics approval was obtained from Renmin University of China. A written informed consent was obtained from the participants and their parents.

### Measures

#### Parental Involvement

In the current study, home-based involvement and academic socialization were measured to reflect parental involvement. Home-based involvement entailed the parental involvement activities in the home with adolescents (e.g., supervising studies and daily life, talking about school life and engaging in activities with them) that improved their school performance (Benner et al., 2016). The following four items were used to assess home-based involvement: (a) on a four-point scale (1 = never, 4 = almost daily), how often did your parents supervise your studies (i.e., checked your homework and gave advice on the problems of homework) in the past week? (b) on a three-point scale (1 = do not care, 3 = very strict), were your parents strict about your daily behavior (i.e., the time you left home to go to school, the time you came back home after school, the time you spent on surfing the Internet and the time you spent on watching TV?) (c) on a three-point scale (1 = never, 3 = often), how often did your parents chat about the following topics with you (i.e., your relationship with your friends, your relationship with your teachers, and incidents that took place in school)? (d) on a six-point scale (1 = never, 6 = more than once a week), how frequently did you and your parents engage in activities together (i.e.., played sports, read books, watched TV, went to museums, and watch sports games)? The mean values of the scales were calculated and then standardized. The Cronbach’s alpha of the items above for the current study is 0.68, which is above 0.60 and is acceptable (Kline, 2000; George and Mallery, 2003). Academic socialization mainly includes parents’ expectations and their faith about their children’s education (Hill and Tyson, 2009; Benner et al., 2016). Therefore, parents’ educational expectation and parents’ confidence in junior school students can be used to measure their academic socialization. To assess academic socialization, students were asked to indicate the years of their parents’ educational expectation and the level of their parents’ confidence in them by using a four-point scale (1 = no confidence, 4 = very confident). The Cronbach’s alpha of the items above for the current study is 0.36. It is appropriate to estimate the inter-item correlation with short scales (Pallant, 2010). Inter-item correlation was 0.22, which was acceptable for the optimal range of inter-item correlation was from 0.20 to 0.40 (Briggs and Cheek, 1986). Confirmatory factor analysis demonstrated that the two-factor model of parental involvement showed acceptable goodness-of-fit index (χ^2^ = 1353.229, *df* = 8, χ^2^*/df* = 169.154, *p* < 0.001, CFI = 0.917, TLI = 0.783, RMSEA = 0.093) (Hu and Bentler, 1999).

#### Junior School Students’ Performance

School performance includes academic achievement and school behavior (Santor et al., 2000). In this study, academic achievement was calculated on the basis of mid-term examination grades of the three main subjects: Chinese, Mathematics, and English. The Cronbach’s alpha of the three examination grades for the current sample is 0.85, which is above 0.60 and is acceptable (Kline, 2000; George and Mallery, 2003). The data of the examination grades was collected directly from schools’ administrations and was standardized. School behavior means the manner of acting in school, such as school attendance and trouble avoidance (Bowen and Bowen, 1999). Similarly, in current study, the school behavior of junior school students were estimated by four items (i.e., “I am seldom late for classes”, “I seldom skip classes”, “I am easy to get along with”, and “My teacher often praises me”) on a four-point scale (1 = completely disagree, 4 = completely agree). The first two items were related to the school attendance, and the latter two items were related to trouble avoidance. The mean value of the four items were standardized. The Cronbach’s alpha of the scale in the current sample is 0.67, which is above 0.60 and is acceptable (Kline, 2000; George and Mallery, 2003). Similarly, a confirmatory factor analysis demonstrated that the two-factor model of parental involvement showed acceptable goodness-of-fit index (χ^2^ = 3110.881, *df* = 13, χ^2^*/df* = 239.299, *p* < 0.001, CFI = 0.924, TLI = 0.837, RMSEA = 0.111) (Hu and Bentler, 1999).

#### Socioeconomic Status

According to Ingels et al. (2005), socioeconomic status was measured by a composite variable based on the parents’ educational attainment, household income, and parents’ occupational prestige (Bradley and Corwyn, 2002; Noble et al., 2012; Jiang et al., 2018). The Cronbach’s alpha of the scale in the present study is 0.63, which is above 0.60 and is acceptable (Kline, 2000; George and Mallery, 2003).

### Data Analysis

Primarily, as the items described in the above section are not in the same range of scales, all data was standardized before analysis. The pairwise method was adopted to handle missing data. The descriptive statistics and correlation matrix were calculated. Secondly, hierarchical regressions were performed using the entry method to explore the roles of parental involvement and SES on junior school students’ performance.

Secondly, four hierarchical regressions were constructed. In the first and second hierarchical regression, academic achievement acted as the dependent variable; in the third and fourth hierarchical regression, school behavior acted as the dependent variable. In the first hierarchical regression, the demographic variables (i.e., sex and age; coded: 1 = male, 0 = female) were entered in step 1, followed by home-based involvement in step 2 and academic socialization in step 3, and SES in step 4. In the second hierarchical regression, the demographic variables were entered in step 1, followed by academic socialization in step 2 and home-based involvement in step 3, and SES in step 4. In the third hierarchical regression, the demographic variables were entered in step 1, followed by home-based involvement in step 2 and academic socialization in step 3, and SES in step 4. In the fourth hierarchical regression, the demographic variables were entered in step 1, followed by academic socialization in step 2 and home-based involvement in step 3, and SES in step 4.

Thirdly, the moderation effect was examined using Model 1 in PROCESS macro (Hayes, 2013). A total of four models were constructed with the moderator of socioeconomic status (M). In Model 1, home-based involvement was set as predictors (X) and academic achievement as outcome (Y); in Model 2, academic socialization was set as predictors (X) and academic achievement as outcome (Y); in Model 3, home-based involvement was set as predictors (X) and school behavior as outcome (Y); in Model 4, academic socialization was set as predictor (X) and school behavior as the outcome (Y). Finally, the simple slope tests were conducted to further validate the moderation effects.

## Results

The results of the descriptive and correlation analyses are shown in **Table 1**. Both home-based involvement and academic socialization were positively related to school behavior (*r* = 0.25–0.33, *p* < 0.001). The association between home-based involvement and academic achievement was insignificant. Academic socialization was positively related to academic achievement (*r* = 0.20, *p* < 0.001). Socioeconomic status was positively related to parental involvement (*r* = 0.23–0.26, *p* < 0.001) and junior school students’ performance (*r* = 0.07–0.19, *p* < 0.001).

**Table 1:** Correlation statistics.

	Descriptive		Pearson Correlation				
	M	*SD*	1	2	3	4	5
1. Home-based involvement	0.02	0.71	–				
2. Academic socialization	0.00	0.78	0.370^∗∗∗^	–			
3. Socioeconomic status	0.01	0.76	0.264^∗∗∗^	0.230^∗∗∗^	–		
4. Academic achievement	0.00	8.77	0.023	0.204^∗∗∗^	0.073^∗∗∗^	–	
5. School behavior	0.00	1.00	0.330^∗∗∗^	0.251^∗∗∗^	0.192^∗∗∗^	0.172^∗∗∗^	–

*^∗∗∗^*p* < 0.001.*

Hierarchical regressions were shown in **Table 2** and **Table 3**. All regression equations were statistically significant (*F* > 68.21, *p* < 0.001). Home-based involvement had a significant explained variance to junior school student’s school behavior (*t* = 31.44, *p* < 0.001). As home-based involvement was not related to academic achievement in the correlation matrix, the negative influence of home-based involvement on academic achievement was spurious in the regression equations. Academic socialization had significant explained variance to academic achievement (*t* = 26.56, *p* < 0.001) and school behavior (*t* = 21.46, *p* < 0.001). SES was entered in step 4 in each regression equation, and the results showed that SES had significant explained variance to junior school students’ performance (*t* > 5.29, *p* < 0.001). These results proved that home-based involvement positively affected school behavior; academic socialization and SES positively affected junior school students’ performance.

**Table 2:** Hierarchical regressions of demographic variables, home-based involvement, academic socialization, socioeconomic status and academic achievement.

	Dependent variable: Academic achievement							
	Step 1		Step 2		Step 3		Step 4	
	*β*	*t*	*β*	*t*	*β*	*t*	*β*	*t*
Constant		12.28***		12.02***		-5.01***		-5.50***
Sex	–0.24	-29.65***	-0.24	-29.65***	-0.24	-30.80***	-0.24	-30.98***
Age	–0.07	-8.84***	-0.07	-8.68***	0.08	7.87***	0.082	8.35***
Home-based involvement			-0.00	-0.31	-0.07	-8.23***	-0.08	-9.14***
Academic socialization					0.28	26.94***	0.27	26.56***
Socioeconomic status							0.044	5.29***
*R^2^*		0.06		0.06		0.11		0.11
*F*		487.54***		325.04***		437.54***		356.29***
Δ*R^2^*				0.00		0.05		0.00
Δ*F*				0.10		725.69***		28.01***
Constant		12.28***		-5.13***		-5.01***		-5.50***
Sex	–0.24	-29.65***	-0.24	-30.55***	-0.24	-30.80***	-0.24	-30.98***
Age	–0.07	-8.84***	0.08	7.95***	0.08	7.87***	0.08	8.35***
Academic socialization			0.25	25.59***	0.28	26.94***	0.27	26.56***
Home-based involvement					-0.07	-8.23***	-0.08	-9.14***
Socioeconomic status							0.04	5.29***
*R^2^*		0.06		0.11		0.11		0.11
*F*		487.54***		558.19***		437.54***		356.29***
Δ*R^2^*				0.04		0.00		0.00
Δ*F*				654.97***		67.77***		28.01***

*^∗^*p* < 0.05, ^∗∗^*p* < 0.01, ^∗∗∗^*p* < 0.001.*

**Table 3:** Hierarchical regressions of demographic variables, home-based involvement, academic socialization, socioeconomic status, and school behavior.

	Dependent Variable: School behavior							
	Step 1		Step 2		Step 3		Step 4	
	*β*	*t*	*β*	*t*	*β*	*t*	*β*	*t*
Constant		9.07***		-0.26		-12.68***		-13.77***
Sex	–0.07	-8.53***	-0.07	-8.28***	-0.07	-8.77***	-0.07	-9.14***
Age	–0.06	-7.67***	0.01	1.49	0.13	13.75***	0.14	14.82***
Home-based involvement			0.34	42.05***	0.28	34.29***	0.27	31.44***
Academic socialization					0.22	22.16***	0.22	21.46***
Socioeconomic status							0.09	11.44***
*R^2^*		0.01		0.12		0.15		0.16
*F*		68.21***		640.34***		619.43***		526.23***
Δ*R*^2^				0.11		0.03		0.01
Δ*F*				1767.78 ***		491.09***		130.96***
Constant		9.07***		-11.73***		-12.68***		-13.77***
Sex	–0.07	-8.53***	-0.07	-9.16***	-0.07	-8.77***	-0.07	-9.14***
Age	–0.06	-7.67***	0.13	12.90***	0.13	13.75***	0.14	14.82***
Academic socialization			0.32	32.52***	0.22	22.16***	0.22	21.46***
Home-based involvement					0.28	34.29***	0.27	31.44***
Socioeconomic status							0.09	11.44***
*R^2^*		0.01		0.08		0.15		0.16
*F*		68.21***		401.24***		619.43***		526.23***
Δ*R^2^*				0.07		0.07		0.01
Δ*F*				1057.23 ***		1175.48 ***		130.96***

*^∗^*p* < 0.05, ^∗∗^*p* < 0.01, ^∗∗∗^*p* < 0.001.*

**FIGURE 1:**
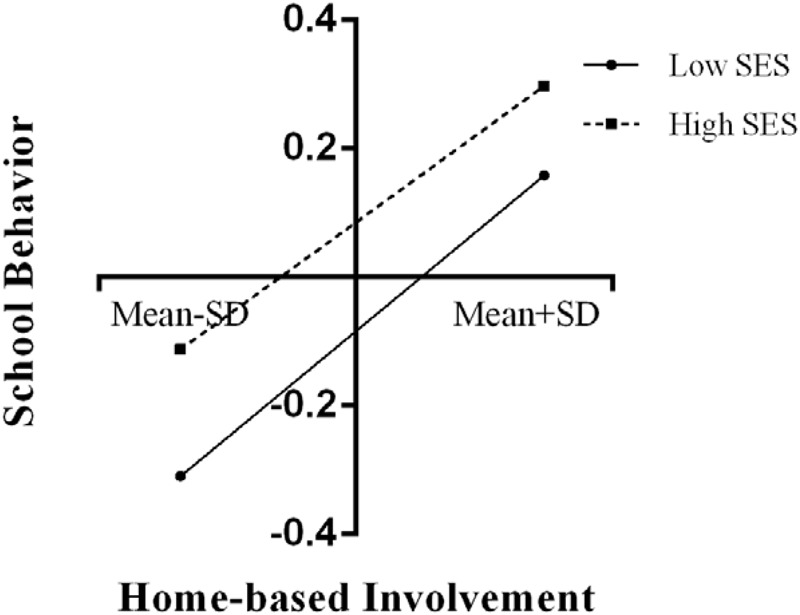
Relation between home-based involvement and school behavior by SES.

**FIGURE 2:**
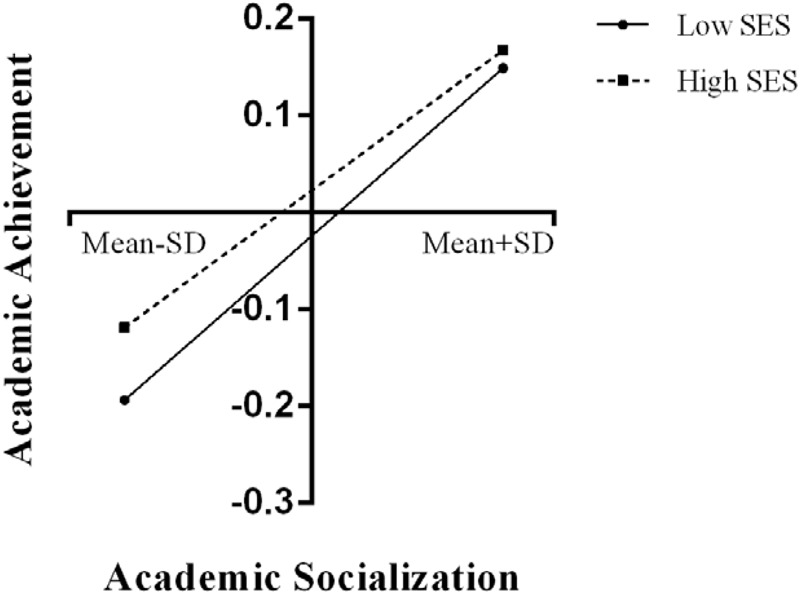
Relation between academic socialization and academic achievement by SES.

The results of moderating analyses are summarized in **Table 4**, showing that socioeconomic status negatively moderated the relationship between home-based involvement and school behavior, as well as the relationship between academic socialization and academic achievement. Other interactions were insignificant. In order to further examine the simple slope effects, we tested two significant two-way interactions that contained the conditional links between parental involvement and junior school students’ performance by SES. In **Figure 1**, home-based involvement was stronger relevant to school behavior in low-SES families (β = 0.34, *p* < 0.001), whereas home-based involvement was weaker relevant to school behavior in high-SES junior school students (β = 0.29, *p* < 0.001). In **Figure 2**, academic socialization was stronger relevant to academic achievement for low-SES junior school students (β = 0.22, *p* < 0.001), whereas academic socialization was weaker relevant to academic achievement for high-SES junior school students (β = 0.18, *p* < 0.001).

**Table 4:** Moderation analysis.

	Coeff	SE	*t*	*p*	LLCI	ULCI
		**Model 1 (Academic Achievement)**				
SES	0.69	0.09	7.27	<0.001	0.50	0.88
HBI	0.08	0.10	0.76	0.449	-0.12	0.27
HBI × SES	0.01	0.13	0.06	0.953	-0.24	0.26
		**Model 2 (Academic Achievement)**				
SES	0.28	0.09	3.13	0.002	0.10	0.45
AC	2.18	0.08	25.87	<0.001	2.02	2.35
AC × SES	-0.30	0.11	-2.87	0.004	-0.51	-0.10
		**Model 3 (School Behavior)**				
SES	0.13	0.01	12.83	<0.001	0.11	0.15
HBI	0.43	0.01	39.06	<0.001	0.41	0.45
HBI × SES	-0.05	0.01	-3.23	0.001	-0.07	-0.02
		**Model 4 (School Behavior)**				
SES	0.18	0.01	17.99	<0.001	0.16	0.20
AC	0.28	0.01	28.72	<0.001	0.26	0.29
AC × SES	-0.01	0.01	-0.46	0.644	-0.03	0.02

*SES, socioeconomic Status; HBI, home-based involvement; AC, academic socialization.*

## Discussion

This study aims to identify the different moderating roles of SES in the relationship between parental involvement and junior school student performance in Chinese culture by replicating and extending previous findings in American culture (Jaeger, 2011; Wang and Sheikh-Khalil, 2014). The results demonstrated that SES negatively moderated both the relationship between academic socialization and academic achievement, and that between home-based involvement and school behavior. The findings imply that parental involvement activities are highly beneficial for children and junior school students in families with low SES. Academic socialization is generally associated with academic success, and home-based involvement closely relates to school behavior.

However, the moderating effect of SES between academic socialization and academic achievement is inconsistent with the findings of Benner et al. (2016), which implies a stronger relationship between academic socialization and academic performance in high-SES families. Benner et al. (2016) claimed that the concerted cultivation of high-SES families contributes to better adolescents’ academic performance. With concerted cultivation, parents provide children with a more advantageous involvement (Lareau, 2003). However, the parenting styles may be different between the United States and China (Lau et al., 2011). Concerted cultivation not only affects the improvement of adolescent school performance, but also improves the other social skills of adolescents (Lareau, 2003). Compared with American parents, Chinese parents pay more attention to learning-related involvement with their children, which results in valuing educational achievement over social functioning (Pomerantz et al., 2014). Moreover, with fewer educational resources, children in low-SES families face more environmental stresses and greater challenges (Shumow et al., 1999). Therefore, parenting practice is particularly crucial for children in low-SES families in China (Wang et al., 2016), and the result of the current study can be interpreted within the current situation in China. Also, the results of the current study were consistent with some of the findings in some aspects (Jaeger, 2011; Roksa and Potter, 2011; Wang and Sheikh-Khalil, 2014), which can also be explained by concerted cultivation in Chinese culture. Youths from low-SES family benefited more from parental involvement in concerted cultivation, which can reduce the gap with youths from high-SES families (Lareau, 2003).

Compared with earlier studies, the current study provides more evidence and proves that concerted cultivation exists in different cultural environments. The negative moderating effects of SES between home-based involvement and junior school student’s school behavior were examined, which was a relatively new finding. Besides, compared with some western studies, the current study mainly focuses on the home-based involvement and academic socialization, providing reasonable results which fit the Chinese parenting culture.

In summary, junior school students in families with low socioeconomic status gain numerous benefits from parental involvement activities. Academic socialization is generally more associated with academic success, and home-based involvement is more closely related to school behavior in low-SES families.

The current study raises important implications for Chinese family education and family intervention policies. Based on the results of the current study, it is known that the intervention program should focus on low-SES families to improve parental involvement, especially for low-SES families, since junior school students benefit more from home-based involvement in low-SES families in China. Secondly, as school-based involvement is less popular in Chinese culture (Lau et al., 2011), the school intervention should promote communication between the school and family to improve school-based involvement, especially for low-SES families in order to increase adolescent school performance.

Some limitations of the current study should be noted. Firstly, there were limitations on the measures of parental involvement. Data for parental involvement with more comprehensive measures are needed to promote the current study. There were validated measurements in the prevailing literature, such as the measurement in the research of Wang and Sheikh-Khalil (2014) or Day and Dotterer (2018). However, as a national representative investigation, the number of items should be taken into account. Future studies may adopt other measurements to further verify the current results. Secondly, the current study only focused on the junior school students’ performance in school, while various aspects of junior school students’ development were exclusive, such as mental health. Thirdly, most of the variables in the model were transformed into standardized z-scores. Therefore, the incremental of variance in regression analysis might be relatively low. Fourthly, various factors should be kept in consideration as predictors of academic achievement in future research. Social cognition, or Theory of Mind (ToM) develops during the child and adolescence period (Bosco et al., 2014), influencing adolescents’ development in many aspects (Brizio et al., 2015). Therefore, ToM is one of the considerable predictors for future research on adolescent school performance. Fifthly, as the cooperation between school and family improves in China, future studies should focus on school-based involvement as well. In addition, the national sample only included junior school students rather than adolescents at all ages. Only one year of data was included in the model. Panel data should be used, and the long-term effects of parental involvement should be discussed in subsequent studies.

## Author Contributions

WD administered the project and contributed to all steps of the work. WD and YG interpreted the data and drafted the manuscript. WD reviewed the manuscript and YG revised it based on the critical comments provided by WD. WD and HB finalized the final submission.

## Conflict of Interest Statement

The authors declare that the research was conducted in the absence of any commercial or financial relationships that could be construed as a potential conflict of interest.
